# Evaluation of the first U.S. staple foods ordinance: impact on nutritional quality of food store offerings, customer purchases and home food environments

**DOI:** 10.1186/s12966-019-0818-1

**Published:** 2019-09-18

**Authors:** Melissa N. Laska, Caitlin E. Caspi, Kathleen Lenk, Stacey G. Moe, Jennifer E. Pelletier, Lisa J. Harnack, Darin J. Erickson

**Affiliations:** 1 School of Public Health, Division of Epidemiology and Community Health, University of Minnesota, 1300 S. 2nd Street, Suite 300, Minneapolis, MN 55454 USA; 2 Department of Family Medicine and Community Health, University of Minnesota, Minneapolis, USA

**Keywords:** Food access, Nutrition, Obesity, Food policy

## Abstract

**Background:**

Many lower-income and racially diverse communities in the U.S. have limited access to healthy foods, with few supermarkets and many small convenience stores, which tend to stock limited quantities and varieties of healthy foods. To address food access, in 2015 the Minneapolis Staple Foods Ordinance became the first policy requiring food stores to stock minimum quantities and varieties of 10 categories of healthy foods/beverages, including fruits, vegetables, whole grains and other staples, through licensing. This study examined whether: (a) stores complied, (b) overall healthfulness of store environments improved, (c) healthy customer purchases increased, and (d) healthfulness of home food environments improved among frequent small store shoppers.

**Methods:**

Data for this natural (or quasi) experiment were collected at four times: pre-policy (2014), implementation only (no enforcement, 2015), enforcement initiation (2016) and continued monitoring (2017). In-person store assessments were conducted to evaluate food availability, price, quality, marketing and placement in randomly sampled food retailers in Minneapolis (*n* = 84) and compared to those in a nearby control city, St. Paul, Minnesota (*n* = 71). Stores were excluded that were: supermarkets, authorized through WIC (Special Supplemental Nutrition Program for Women, Infants, and Children), and specialty stores (e.g., spice shops). Customer intercept interviews were conducted with 3,039 customers exiting stores. Home visits, including administration of home food inventories, were conducted with a sub-sample of frequent shoppers (*n* = 88).

**Results:**

Overall, findings indicated significant improvements in healthy food offerings by retailers over time in both Minneapolis and St. Paul, with no significant differences in change between the two cities. Compliance was low; in 2017 only 10% of Minneapolis retailers in the sample were fully compliant, and 51% of participating Minneapolis retailers met at least 8 of the 10 required standards. Few changes were observed in the healthfulness of customer purchases or the healthfulness of home food environments among frequent shoppers, and changes were not different between cities.

**Conclusions:**

This study is the first evaluation a local staple foods ordinance in the U.S. and reflects the challenges and time required for implementing such policies.

**Trial registration:**

NCT02774330.

## Introduction

The Centers for Disease Control and Prevention, Institute of Medicine, and other health authorities have identified improving access to healthy foods as a strategy for local governments to help prevent obesity [1–3]. Many communities, particularly lower-income and racially diverse communities, have limited access to healthy foods [4]. Numerous studies indicate that supermarkets are more likely to locate in high-income and low-minority areas, and convenience stores are more likely to locate in low-income and high-minority areas [4]. As a result, disparities in access may contribute to health disparities, in that supermarkets generally offer a wider variety of healthy, high-quality foods, and small convenience stores carry higher-calorie, processed foods at higher prices [4–6].

Over the past decade, there has been increasing attention on strategies for increasing healthy food availability in venues such as convenience stores, or “corner stores,” as a mechanism for health promotion [1, 2, 7, 8]. To date, research evaluating healthy corner store programming (i.e., technical assistance programs working one-on-one with retailers) has yielded mixed results. When evaluating program effects on healthy food availability in stores and customers’ psychosocial characteristics, such as self-efficacy, knowledge, and attitudes about healthy eating, many published interventions have found significant positive effects, [9–13] though not all have detected positive effects [14] and psychosocial factors showing improvements have not necessarily been consistent across the literature [7]. In addition, some studies have shown beneficial effects on healthy food purchasing and/or consumption, [10, 12, 13, 15] while others have not [16, 17]. Importantly, most interventions have been time- and resource-intensive, creating challenges for sustainability; [18] moreover, participation in such programs is voluntary, and therefore includes retailers particularly capable and/or motivated to sell healthy food. Policy initiatives could expand these types of efforts by broadening reach, providing incentives for stores to stock healthy foods, and enforcing healthy standards. Although policy work in this area has been limited, one potential action area is around the implementation of health criteria, such as minimum stocking requirements for healthy foods, as a condition of business licensing [19].

The City Council of Minneapolis, MN passed a Staple Foods Ordinance in 2008 requiring licensed grocery stores to carry staple foods, including three varieties each of (a) breads or cereals, (b) dairy products and (c) meat, poultry or fish. Five varieties of fresh fruits or vegetables were also required [20]. This was the first and is still one of the only policies of its kind. It was originally passed, in part, as a crime prevention policy, targeting retailers that offered little food and were locations for criminal activity. However, it had loopholes limiting its capacity to improve food access. For example, the original ordinance specified no minimum quantities for stocking (only minimum varieties), and food types were not subject to health criteria (for example, not requiring whole grain breads or low-fat dairy). Therefore, in a continued effort to address healthy food access, the City of Minneapolis significantly revised its Staple Foods Ordinance in 2014.

Revisions included improvements to align the policy with the Dietary Guidelines for Americans and stocking requirements for retailers participating in the Special Supplemental Nutrition Program for Women, Infants and Children (WIC) [21, 22]. Thus, stocking requirements increased notably across an array of nutrient-rich foods that are low in energy density. Minimum stocking requirements were created for 10 product categories, including fruits and vegetables, whole grain rich products, and low-fat dairy. In addition, to count toward the revised ordinance standards, perishable items like produce needed to be offered “in good condition, not overripe or seriously deformed and free from decay, discoloration, bruising and surface damage” [23]. All required product types and minimum quantities are detailed in Table 1.

**Table 1:** Minimum stocking standards set forth by the Minneapolis Staple Food Ordinance, 2015

Category	Specifications
Fruit/Vegetables	• 30 lbs. or 50 items fresh and/or frozen • At least 7 varieties; at least 5 must be fresh • No more than 50% from a single variety • No added ingredients (including syrups, dips or cheese)
100% juice	• 6 containers of 100% juice; at least 2 must be citrus • Frozen/non-frozen concentrate: 11.5–12 oz. containers • Juice: 59 oz. or larger containers
Whole grain cereal	• 4 boxes or bags, 12 oz. or larger, whole grain cereal or cereal grains • At least 3 varieties
Other whole grains	• 5 pounds • At least 3 varieties such as bread, corn tortillas, brown rice or oatmeal • No popcorn with salt and/or added fat
Milk	• 5 gal unsweetened, unflavored • Gallon or half-gallon containers • At least 2 of the following varieties: skim/ nonfat, 1%, or 2% milk, or “plain” or “original” soy milk or other milk alternatives
Eggs	• 6 one dozen containers • Large size only
Cheese	• 6 pounds • Packages of at least 1 half pound (8 oz.) • At least 3 varieties • Does not include processed cheese products
Dried peas, beans and lentils	• 4 packages • Up to 16 oz. in size • No added ingredients or seasonings.
Canned beans	• 192 oz. total of canned beans or legumes • At least 3 varieties • No added fats or meats; no baked beans or chili beans.
Meat, fish, and other proteins	• At least 3 varieties of meat, poultry, canned fish packed in water, or vegetable proteins such as nut butter and/or tofu. • Nut butter up to 18 oz.; may not contain other products such as jelly

Implementation of the revised Staple Foods Ordinance began in April 2015. Minneapolis City Council initiated a one-year period (April 1, 2015 – March 31, 2016) for implementation with no enforcement. This period was added to the policy timeline in response to concerns about retailers’ abilities to comply. Implementation by the Minneapolis Health Department during this year included: (1) visits to all eligible retailers (except supermarkets, which were contacted via telephone), including compliance assessments and retailer education on requirements; (2) group trainings and individual consultations offered to retailers on product procurement, marketing and merchandizing; (3) free resources for retailers, including in-store merchandising kits and promotional supplies, and low-interest loans for infrastructure/equipment enhancements; and (4) meetings with corporate chain store representatives, and presentations to local business associations about the ordinance. The Healthy Living Team at the health department also continued visiting all stores at least once per year, providing one-on-one education for retailers as needed after the implementation-only period ended in 2016.

In 2016, enforcement began using the city government’s standard enforcement procedures including inspection and, in cases of non-compliance, giving health inspectors the authority to utilize step-wise consequences of warning letters, citations, and fines for non-compliance. Compliance checks are included in all routine health inspections, which occur every 1–2 years. If retailers are found to be non-compliant, health inspectors may issue to retailers a violation notice. Further, if retailers remain non-compliant upon a 30–60 day re-inspection by the health inspector, formal citations and fines may be issued, and ultimately business licenses could be revoked. Between April 2017 and April 2018, 118 violations of the Staple Foods Ordinance were written for 76 stores, but no fines were issued.(K. Klinger, personal communication).

The purpose of this study was to evaluate the Minneapolis Staple Foods Ordinance. Specifically, we examined whether: (a) stores complied, (b) overall healthfulness of store environments improved, (c) healthy customer purchases increased, and (d) healthfulness of home food environments improved among frequent small store shoppers. We hypothesized that retailers would comply and that the ordinance would have a positive impact on healthfulness of store environments, customer purchases and home food environments.

## Methods

The STORE (STaple foods ORdinance Evaluation) Study was designed to gather data at four times: pre-policy revisions (July–December 2014, hereafter called time 1), during the implementation-only phase (i.e., no enforcement; September–October 2015, time 2), at the initiation of enforcement (May–July 2016, time 3), and after continued monitoring (August–December 2017, time 4). Our work compared changes in stores in Minneapolis to those in St. Paul, MN, a similarly-sized adjacent city with no such ordinance (i.e., control city).

### Store sample

We identified stores through government lists of stores with grocery licenses. Stores were ineligible for the evaluation if they: (1) were supermarkets; (2) were WIC-authorized (because they were presumed to already meet minimum requirements); (3) had invalid licensing addresses; or (4) were exempt from the ordinance. Ordinance exemptions include retailers with ≤100 ft^2^ retail space, small vendors in market areas (e.g., produce stands), liquor or specialty stores (e.g., spice shops), and stores in the downtown commercial district. Stores in St. Paul that met these criteria were also considered ineligible for evaluation through the study.

**Fig. 1:**
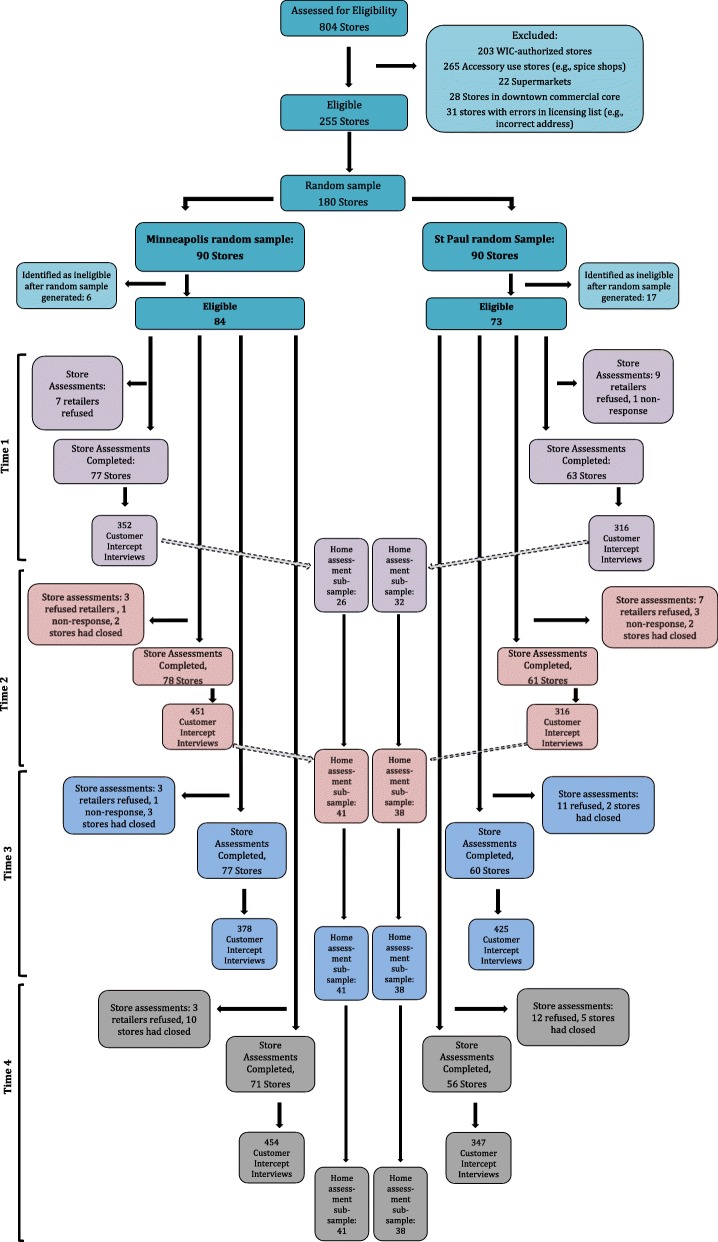
Flow of participants for store assessments and customer intercept surveys at each data collection time point of the study (2014–2017)

Of 255 eligible stores, 180 (90 per city) were randomly selected. After visiting stores prior to data collection, 20 additional stores were deemed ineligible due to new participation in WIC (*n* = 5), misclassification of supermarkets and/or exempt stores on license listings (*n* = 10), and going out of business by the time of our store visit (n = 5). Of the remaining 160 retailers, 159 actively gave consent and participated in the study at one or more of the four data collection time points. (See Fig. 1 for more detail on participation at each time point.)

For the collection of data via the store assessments and customer intercept interviews (described in detail below), permission to collect these data was obtained from a store employee each time research staff visited the store. Store employees received no additional compensation for allowing this data collection to occur. Store managers received a $75 gift card at each data collection time point for participating in a manager interview (data not presented in this manuscript). They gave verbal consent prior to participating in the interview at each time point, and were given a Participant Information Sheet with additional information about the study.

### Store assessments

Store environments were assessed using a modified instrument from the Yale Rudd Center [24]. The Rudd Center instrument is similarly structured to the validated Nutrition Environment Measure Survey in Stores (NEMS-S) instrument [6, 25]. Both have lists of items in specific package sizes for which availability, price, and (for fresh fruits/vegetables) quality is recorded. In this current study, the list of items was adapted to align with the ordinance requirements [26, 27]. The adapted instrument, along with other data collection tools used in the STORE study, is available at: https://conservancy.umn.edu/handle/11299/203078.

Our store assessment evaluated the availability and price of 69 items, including fresh, frozen and canned fruits and vegetables with no added ingredients (other than salt in canned products), 100% juice, whole grain-rich bread, whole-wheat or corn tortillas, brown rice, whole grain-rich cereals in packages ≥12 oz., low-fat milk/milk substitutes, dry beans and lentils in packages ≤16 oz., cheese in packages ≥8 oz., eggs in dozen containers, plain nut butters in ≤18 oz. containers, canned fish in water, and tofu, as well as some less healthy comparison items (e.g., white bread, whole milk). It also addressed *varieties* of milk; fresh, frozen and canned fruits and vegetables; cheese; canned beans; whole grain-rich cereals; whole grain-rich bread; brown rice; and whole grain tortillas as well as the *quality* of twenty fresh fruits and vegetables.

Data were used to create a Healthy Food Supply (HFS) score (a primary outcome for this study) by summarizing availability, price, quality, and varieties available [24]. The HFS score had a possible range of 0–31, where higher scores indicate a healthier store inventory [24, 26]. It is based on a similar NEMS-based score [5, 28, 29]. In addition, policy compliance was calculated for each of 10 product categories in the ordinance, as well as whether retailers met all ordinance standards (10/10 product categories), ≥80% of standards and/or ≥ 60% of standards.

To describe stores, we collected information on store type, number of aisles and cash registers, and whether the store was an authorized retailer through the federal food assistance program, SNAP (Supplemental Nutrition Assistance Program), meaning that the retailer accepted SNAP benefits as a form of payment.

### Customer intercept interviews

To evaluate nutritional quality of food/beverage purchases, we conducted intercept interviews with shoppers exiting stores. Details on development, adaptation and implementation of customer intercept methods have been published elsewhere [30]. Briefly, while standing outside near the store exit, data collectors invited customers who appeared ≥18 years old and had a bag or a visible food/beverage purchase to participate in the interview. After verifying eligibility and obtaining consent, data collectors recorded participants’ food and beverage purchases (quantity, size, product name, and price paid) and administered a 5-min structured interview, which included questions addressing customers’ shopping frequency at that store. The survey concluded with participants’ demographic information and self-reported height and weight.

For completion of the interview, participants were offered a $10 gift card. The timing and number of interviews completed are depicted in Fig. 1. Overall response rate was 35%, with significantly higher response rates at corner stores/convenience stores/small groceries (47%) and dollar stores (46%), versus food-gas marts (32%) and pharmacies (26%). The distribution of race/ethnicity, but not gender, differed between participants and non-participants (*p* < 0.01), with greater participation among those identified as Black versus White [30].

### Food and nutrient analyses

Data on purchased foods and beverages were entered by trained staff into the Nutrition Data System for Research (NDSR), a software application developed at the University of Minnesota Nutrition Coordinating Center that generates values for nutrients and food servings for numerous product categories [31]. For participant purchases, measures included whether the participant purchased ≥1 serving in each food/beverage category (e.g., ≥1/2 cup fruits/vegetables) and nutrient content of the purchase, including calories, added sugars (% total calories), saturated fatty acids (% total calories), and sodium (mg/1000 cal). Energy density was also calculated by dividing calories by gram weight [32]. Because energy density is accurately calculated using only food items, beverages were not included in energy density analyses [32].

We computed a HEI-2010 score by summing 12 subcomponents to create a score with a range of 0–100 [33]. The HEI-2010, updated from HEI-2005, [34] is a validated tool measuring consistency with federal dietary guidelines. Similar to HEI-2005, HEI-2010 is one of the most widely used dietary composite scores in the scientific literature, and has been shown to: produce a distribution of scores to detect meaningful inter-individual variations in diet, differentiate between sub-groups with known difference in dietary quality (i.e., men vs. women, older vs. young adults, smokers vs. non-smokers), have limited collinearity with caloric intake, and have internal consistency. These results have been reported in depth by Guenther and colleagues [35, 36].

### Home assessments

In the intercept interviews outside of stores, participants were asked “how often do you shop at this store?,” with 8 possible frequency response options ranging from “more than once a day” to “less than once a month.” Those who reported shopping at the store at least once per week or more were additionally invited to participate in a longitudinal sub-study that included an objective home food environment assessment, interviews and questionnaires completed in participants’ homes at a later date. Participants were recruited from both Minneapolis and St. Paul stores at baseline, and interested customers provided their contact information to study staff for future follow up. Participants were contacted to complete home assessments at each of the four time points. Due to insufficient recruitment in the longitudinal sub-study at baseline (*n* = 56), we enrolled 32 additional participants at time 2 using the same methods and invited them to participate in home visits again at times 3 and 4. In total, we retained 74% of our sample by time 4.

In teams of two, trained study staffers visited participants’ homes and completed an assessment of the home food environment at each time point using a previously validated tool [37]. The Home Food Inventory (HFI) included approximately 200 items across 13 categories and were in a checklist format with yes/no (1/0) response options, indicating whether the items were present in the home. Higher scores represented greater availability. In addition, staff recorded whether the vegetable, fruit, and bread items were fresh, frozen, dried or canned, as appropriate. Foods in the dairy, added fats, frozen desserts, prepared desserts, and savory snacks categories were categorized into regular-fat or reduced-fat groupings; beverages were categorized into regular sugar and low sugar categories; and foods in the two ready-access categories were further sub-grouped into more healthful and less healthful categories. Although the categorization of foods into healthful and less healthful categories is not straightforward, the HFI is used to assess each food by its typical fat and sugar content when determining its category. To assess the overall obesogenic home food availability, a summative score [37] was created that includes regular-fat versions of cheese, milk, yogurt, other dairy, frozen desserts, prepared desserts, savory snacks, added fats; regular-sugar beverages; processed meat; high-fat quick, microwavable foods; candy; and access to unhealthy foods in refrigerator and kitchen. The obesogenic home food availability score potential range was from 0 to 71. In addition, we created summative scores for vegetables, fruit, reduced-fat milk and whole grain breakfast cereal in order to align with the stocking requirements in the Staple Foods Ordinance.

During the home visits, participants also responded to interviewer administered and self-reported survey items related to sociodemographic factors, shopping habits, and food choice. For the purposes of this analysis, these questions included: number of people living in the household; age, sex, race/ethnicity, educational attainment, employment status, frequency of purchasing food and beverages at the store where they completed their customer intercept survey (and were recruited for the study), and self-reported height and weight.

### Neighborhoods

Using store locations, we considered area-level socio-demographic characteristics (i.e., poverty, racial/ethnic composition) as potential confounders of city differences over time. Data were drawn from 5-year American Community Survey estimates (ACS, 2009–2015) [38]. Data were attributed to stores based on Census tract location.

### Power calculation

A priori target sample sizes were based on a number of power and sample size calculations and included ranges incorporating attrition, various detectable effect sizes, and a mix of outcomes and variance estimates. Original power calculations suggested that detectable differences at the store level would be less than .2 SD units when analyzing two or more time points. Detectable differences over 2 or more time points in calories purchased by customers would be approximately .17 SD, and detectable differences over 2 or more time points in fruit/vegetable availability at the household level would be approximately .5 SD. The sample sizes that were recruited for customer and household levels exceeded a priori targets. The final sample size for stores was slightly less than originally targeted, resulting in detectable differences over time that increased from approximately .2 SD units to approximately .24 SD units.

### Analyses

We calculated pre-policy descriptive statistics separately for Minneapolis and St. Paul. We compared cities using bivariate Chi-square tests for percentages and general linear models for means (*p* < .05).

A priori, we identified two primary outcomes for this natural experiment: HFS score among stores and total calories purchased by customers. To test changes in store-level outcomes, including HFS score and ordinance compliance, we computed mixed model regression analyses. We first tested an overall time by city interaction to examine the effect of the policy over all four time points (i.e., our primary analyses) and then explored secondary analyses of single degree of freedom planned contrasts to test time by city interactions from time 1 to 2, from time 1 to 3, and from time 1 to 4. Models were adjusted for repeated measures and covariates shown to be significantly differ between cities in bivariate comparisons.

To test changes in customer-level outcomes, we computed similar mixed model regression analyses on customer purchasing and home food environment data. For purchasing and home food environment analyses, we included store identification as a random effect due to nesting of customers within stores.

For both store-level and customer purchasing models, we conducted unadjusted post-hoc analyses examining city-specific effects, limiting models to one city. We also conducted the same type of planned contrasts as in main analytic models. All analyses were conducted in SAS (SAS/STAT Version 9.4).

## Results

Table 2 details store- and neighborhood-level characteristics at time 1 (pre-policy revision) for stores in the sample. Most stores were classified as (a) corner stores, convenience stores or small groceries, or (b) food-gas marts. Fewer stores were dollar stores, pharmacies or general retailers. The randomly selected sample yielded various sized stores, as evidenced by the number of aisles and cash registers. Most retailers (> 90%) were SNAP authorized. There were no significant differences in store characteristics by city.

**Table 2:** Store- and neighborhood characteristics at baseline (pre-policy revisions), Minneapolis and St. Paul, MN, 2014 (*n* = 140)

Store characteristics	Minneapolis	St. Paul	*P*-value^b^
	% (N)	% (N)	
Store type			0.49
Corner stores, convenience stores, small groceries	44 (34)	32 (20)	
Food-gas marts	31 (24)	41 (26)	
Dollar stores	9 (7)	10 (6)	
Pharmacies	14 (11)	17 (11)	
General retailers	1 (1)	0 (0)	
Number of aisles in store^d^			0.92
0–4	36 (27)	34 (21)	
5–8	36 (27)	39 (24)	
9+	28 (21)	26 (16)	
Number of cash registers^d^			0.25
1	44 (33)	30 (18)	
2–3	37 (28)	47 (28)	
4+	19 (14)	23 (14)	
SNAP authorized	92 (71)	98 (62)	0.13
Neighborhood characteristics ^a^	Mean (SD)	Mean (SD)	
% Poverty	21.7 (17.3)	19.1 (13.5)	0.35
% < 185 of poverty^c^	36.7 (23.2)	36.7 (20.4)	1.0
% Hispanic	11.3 (10.3)	9.8 (6.5)	0.35
Non-Hispanic			
% White	58.9 (24.3)	52.3 (23.7)	0.11
% Black	18.3 (17.5)	15.7 (14.9)	0.37
% American Indian/Alaskan Native	1.6 (2.6)	0.6 (1.0)	**0.007**
% Asian	5.8 (6.9)	18.1 (12.3)	**< 0.0001**
% Native Hawaiian or other Pacific Islander	0.03 (0.1)	0.1 (0.4)	0.06
% Some other race alone	0.3 (0.6)	0.1 (0.3)	**0.03**
% Two or more races	3.9 (2.1)	3.3 (2.1)	0.08

^a^Based on the census tract where store was located (from American Community Survey; 2009–2013 5-year estimates)

^b^Comparisons between cities; bold indicates *p* < 0.05

^c^Percent of residential households with a household income less than 185% percent of the US Poverty Guidelines (https://aspe.hhs.gov/poverty-guidelines)

^d^Note: Number of missing values (if any) for each variable: number of aisles = 4; number of cash registers = 5

The Census tracts in which participating retailers were located had, on average, 36.7% of residents whose annual household income was ≤185% of the federal poverty line. About half of residents in these areas were non-Hispanic White (58.9% in Minneapolis, 52.3% in St. Paul). By city, there were some differences by race (for American Indian/Alaskan Native, Asian and other race).

Table 3 presents characteristics of customers who participated in intercept interviews by time point. City-specific means for age ranged from 37 to 42 years over time. Samples for each time point were approximately 52–64% non-White. Samples were well-distributed by gender, education and employment. Most customers reported shopping at the store more than once a week. Few significant differences between cities were identified, including differences by race/ethnicity (time 3 only), employment (time 3 only) and prevalence of obesity (BMI ≥30 kg/m^2^; times 2–4).

**Table 3:** Descriptive characteristics of participating customers in intercept surveys, Minneapolis and St. Paul, MN, 2014–2017 (*n* = 3,039)

	Time 1		Time 2		Time 3		Time 4	
	Pre-policy change, 2014		Implementation only, no enforcement, 2015		Early initiation of enforcement, 2016		Continued monitoring, 2017	
	Minneapolis (*n* = 352)	St. Paul (*n* = 316)	Minneapolis (*n* = 451)	St. Paul (n = 316)	Minneapolis (*n* = 378)	St. Paul (*n* = 425)	Minneapolis (*n* = 454)	St. Paul (*n* = 347)
	Mean (SD)							
Age (years)	**39 (15)**	**42 (15)**	37 (14)	39 (14)	39 (14)	38 (14)	**39 (15)**	**42 (15)**
	Percent							
Sex: male	60	52	57	54	59	56	**63**	**53**
Race/ethnicity								
Hispanic	3	4	5	8	**5**	**8**	4	3
Non-Hispanic								
White	48	45	40	41	**38**	**41**	34	42
Black	34	38	37	36	**40**	**36**	40	38
American Indian/Alaskan Native	5	3	5	3	**7**	**3**	6	2
Asian	3	4	4	3	**2**	**5**	3	2
Other race	4	4	4	3	**5**	**4**	6	6
Multi-race	3	4	4	6	**3**	**4**	7	7
Education								
High school diploma or less	37	39	37	34	40	41	39	38
Some college	37	35	37	43	37	35	38	40
Bachelor’s degree or higher	26	25	26	23	23	24	24	22
Employment								
Employed	62	65	67	73	**65**	**72**	64	67
Unemployed/disability	28	23	20	13	**25**	**18**	21	21
Other (student, retired)	10	11	13	14	**10**	**10**	15	12
Frequency of shopping at store								
Less than once a week	25	29	26	24	27	27	25	27
1–6 times a week	45	43	44	47	42	45	41	41
At least once a day	30	28	30	28	31	28	33	32
Weight status								
Overweight (BMI ≥ 25, < 30 kg/m^2^)	30	31	34	34	31	29	38	35
Obese (BMI ≥ 30 kg/m^2^)	32	33	**26**	**34**	**29**	**39**	**30**	**37**

Note: Bold text indicates significant chi-square test (p < .05) between cities within time point

Note: Number of missing values (if any) for each variable for each time point: Time 1: age = 2, sex = 3, race/ethnicity = 3, education = 2, employment = 1, frequency of shopping at store = 1, weight status = 22; Time 2: age = 12, sex = 5, race/ethnicity = 5, education = 7, employment = 7, frequency of shopping at store = 3, weight status = 23; Time 3: age = 10, sex = 3, race/ethnicity = 8, education = 4, employment = 6, weight status = 37; Time 4: age = 12, sex = 1, race/ethnicity = 9, education = 5, employment = 4, frequency of shopping at store = 4, weight status = 34

**Fig. 3:**
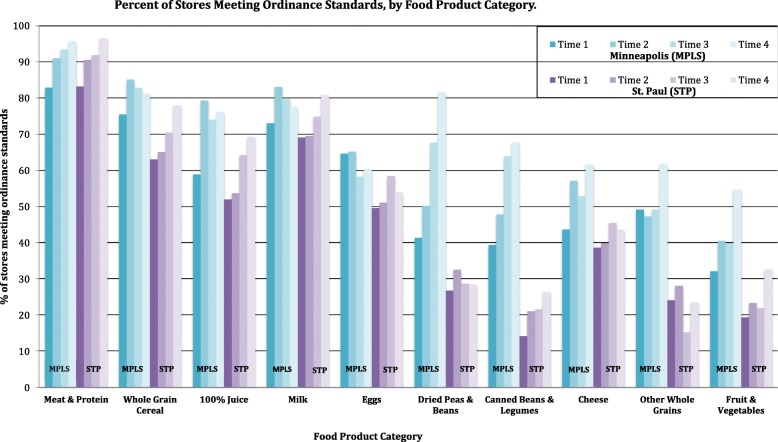
Percent Stores Meeting Ordinance Standards,by Food Product Category

Table 4 presents results of changes in store-level HFS score across times 1–4 (2014–2017) in Minneapolis and St. Paul. Although there were significant overall increases in HFS score by time (*p* = 0.008) and by city (*p* = 0.0007), there was no significant differential change in HFS score over time by city (*p* = 0.99), suggesting no impact of the policy specifically on Minneapolis compared to St. Paul. Furthermore, change in percent of stores meeting ordinance standards did not significantly differ between cities. At time 4 (2017), 9.6% of Minneapolis stores in the sample were fully compliant with the ordinance, and about half (50.5%) met 8 of the 10 standards in the ordinance. The Figure 3 in Appendix 2 shows the percent of stores meeting each of the 10 ordinance standards over time. Analyses of change in compliance with ordinance standards for each of the 10 food/beverage groups did not yield significant differences over time between cities except for dried peas and beans for which a greater increase was observed in Minneapolis in comparison to St Paul (*p* < 0.0001).

**Table 4:** Impact of the Minneapolis Staple Foods Ordinance over time on healthy food availability in stores, 2014–2017 (*n* = 155 stores)

Outcome	City					Overall Effects		
		Time 1	Time 2	Time 3	Time 4			
		Pre-policy change, 2014	Implementation only, no enforcement, 2015	Early initiation of enforcement, 2016	Continued monitoring, 2017	Main effects		Interaction
						Time	City	Time x City
		% (SE)	% (SE)	% (SE)	% (SE)	P(df = 3)	P(df = 1)	P(df = 3)
Primary outcome								
Healthy Food Supply (HFS) score	Minneapolis	10.6 (0.5)	11.0 (0.5)	11.3 (0.4)	11.8 (0.5)	**0.008**	**0.0007**	0.99
	St. Paul	8.7 (0.4)	8.9 (0.5)	9.3 (0.5)	9.7 (0.6)			
	p-net	**–**	*p* = 0.79	*p* = 0.90	*p* = 0.85			
Compliance								
Met all ordinance standards	Minneapolis	7.4 (2.9)	13.7 (3.9)	11.5 (3.6)	9.6 (3.4)	0.22	**0.003**	0.57
	St. Paul	0.4 (0.4)	2.1 (1.7)	1.9 (1.7)	4.0 (2.5)			
	p-net	**–**	*p* = 0.26	*p* = 0.51	*p* = 0.73			
Met ≥80% of ordinance standards	Minneapolis	24.4 (4.9)	40.7 (5.6)	31.0 (5.2)	50.5 (5.9)	**0**.**0003**	**< 0.0001**	0.83
	St. Paul	3.2 (2.2)	11.6 (4.1)	6.7 (3.2)	14.4 (4.7)			
	p-net	**–**	*p* = 0.35	*p* = 0.56	*p* = 0.55			
Met ≥60% of ordinance standards	Minneapolis	52.7 (5.8)	61.1 (5.6)	75.2 (4.9)	76.0 (5.1)	**0.0004**	**0.0002**	0.37
	St. Paul	35.4 (6.1)	39.9 (6.3)	43.7 (6.4)	48.9 (6.7)			
	p-net	**–**	*p* = 0.64	*p* = 0.10	*p* = 0.24			
Met ordinance standards for specific categories:								
*Milk and milk alternatives*	Minneapolis	73.0 (5.6)	83.1 (4.3)	79.6 (4.9)	77.5 (5.4)	0.13	0.42	0.25
	St. Paul	69.1 (6.0)	69.6 (5.8)	74.8 (5.6)	80.8 (5.0)			
	p-net	**–**	*p* = 0.11	p = 0.85	*p* = 0.39			
*Eggs*	Minneapolis	65.6 (5.5)	66.1 (5.4)	59.1 (5.7)	60.5 (5.8)	0.99	0.11	0.37
	St. Paul	50.2 (6.3)	51.9 (6.4)	58.5 (6.4)	53.9 (6.6)			
	p-net	**–**	p = 0.90	*p* = 0.14	*p* = 0.43			
*Cheese*	Minneapolis	46.9 (5.9)	57.2 (5.7)	52.9 (5.7)	61.7 (5.8)	0.21	0.09	0.42
	St. Paul	39.7 (6.3)	41.1 (6.4)	45.4 (6.5)	43.5 (6.7)			
	p-net	**–**	*p* = 0.25	*p* = 0.98	*p* = 0.23			
*100% juice*	Minneapolis	59.0 (5.9)	79.3 (4.7)	74.0 (5.0)	76.1 (5.1)	**0.001**	**0.04**	0.11
	St. Paul	52.0 (6.4)	54.4 (6.5)	64.3 (6.2)	69.3 (6.1)			
	p-net	**–**	*p* = **0.02**	*p* = 0.65	*p* = 0.89			
*Fruit/Vegetable*	Minneapolis	32.0 (5.3)	40.5 (5.5)	39.9 (5.6)	54.7 (5.9)	**0.0007**	**0.009**	0.95
	St. Paul	19.3 (5.0)	23.3 (5.5)	21.8 (5.4)	32.6 (6.4)			
	p-net	**–**	*p* = 0.71	*p* = 0.61	*p* = 0.57			
*Meat and vegetable protein*	Minneapolis	82.9 (4.3)	91.0 (3.3)	93.5 (2.9)	95.8 (2.4)	**< 0.0001**	0.95	0.96
	St. Paul	83.1 (4.7)	92.0 (3.4)	91.9 (3.5)	96.6 (2.4)			
	p-net	**–**	*p* = 0.80	*p* = 0.70	*p* = 0.83	.		
*Canned beans and legumes*	Minneapolis	39.2 (5.6)	48.3 (5.7)	64.7 (5.6)	67.8 (5.6)	**0.0002**	**< 0.0001**	0.42
	St. Paul	14.5 (4.5)	21.8 (5.3)	21.5 (5.3)	26.5 (5.8)			
	p-net	**–**	p = 0.76	*p* = 0.27	*p* = 0.32			
*Dried peas and beans*	Minneapolis	41.2 (5.8)	50.2 (5.9)	67.8 (5.6)	83.0 (4.6)	**< 0.0001**	**< 0.0001**	**< 0.0001**
	St. Paul	26.8 (5.8)	33.9 (6.0)	29.0 (5.7)	28.6 (6.0)			
	p-net	**–**	*p* = 0.94	*p* = **0.01**	p = < **0.0001**			
*Whole grain cereal*	Minneapolis	76.6 (5.0)	85.2 (4.2)	82.8 (4.6)	81.2 (4.7)	0.08	**0.04**	0.38
	St. Paul	63.1 (6.0)	66.7 (6.4)	70.5 (5.8)	78.0 (5.4)			
	p-net	**–**	*p* = 0.37	p = 0.90	*p* = 0.28			
*Other whole grains*	Minneapolis	49.2 (5.7)	47.3 (5.6)	49.2 (5.7)	61.9 (5.8)	0.10	**<.0001**	0.10
	St. Paul	24.3 (5.4)	28.5 (5.8)	15.1 (4.6)	23.4 (5.7)			
	p-net	**–**	*p* = 0.41	p = 0.23	*p* = 0.22			

Note: Models are adjusted for repeated measures over time and for neighborhood race/ethnicity (the only covariate that was significant in bivariate comparisons between Minneapolis and St. Paul at baseline); HFSS is a linear regression model due to a continuous outcome; full compliance is a linear regression model due to zero cell for St. Paul at baseline causing non-convergence in logistic regression model; all others are logistic regression models; p-net values refer to changes in time*city effect from Time1 to Time 2, Time 1 to Time 3, and Time 1 to Time 4 respectively

Note: Number of missing values (if any) for each outcome variable at each time point: Time 1: eggs = 2; cheese = 7, canned beans = 2, dried beans = 1, cereal = 1, grains = 1; Time 2: eggs = 2, cheese = 2, juice = 1, meat = 1, canned beans = 3, dried beans = 2, cereal = 2, grains = 1; Time 3: eggs = 1, canned beans = 1, dried beans = 1; Time 4: dried beans = 2

Bolded values denote *p* < 0.05

Unadjusted city-specific regression models were also examined post hoc to determine if changes in these measures were observable for one or both cities, but not necessarily significantly different between the cities (data not shown). We found statistically significant increases in Minneapolis across 27 of 56 post hoc analyses, with all significant findings in the hypothesized direction. These included increases in HFS score, in stores meeting ≥80% of ordinance standards and ≥ 60% of standards, as well as stores meeting standards for milk, cheese, 100% juice, fruits and vegetables, meats and proteins, canned beans and legumes, and dried peas and beans (though significant differences were not consistent across all time intervals). For St. Paul, we detected significant changes in 15 of 52 post hoc analyses, yielding evidence for increases in staple food availability for all models.

Table 5 details results for changes in customer-level purchasing by city over time. On average, customer purchases included 2–3 items and consisted of 800–1600 cal total, with few purchases including at least one serving of fruit, vegetables, whole grains or skim/reduced fat milk (< 10%). With respect to the primary customer purchasing outcome (calories purchased), there was a decrease in calories purchased in both Minneapolis and St Paul, with no significant difference in change between the two cities (*p* = 0.76 for time x city interaction). Overall there were changes over time in a few of the other measures of the healthfulness of food purchasing, but none of the changes were significantly difference between Minneapolis and St Paul. In unadjusted post hoc city-specific models, several significant within-city changes in customer purchasing were observed in Minneapolis (data not shown), with significant changes across 15 of the 48 models (only 9 of which yielded results in the hypothesized direction). For St. Paul, significant changes were observed across 3 of the 48 models (2 of which were shifts toward more healthful purchasing).

**Table 5:** Impact of the Minneapolis Staple Foods Ordinance over time on customer food/beverage purchasing, 2014–2017 (n = 3,039)

Outcome	City	Assessment Period				Overall effects		
		Time 1	Time 2	Time 3	Time 4			
		Pre-policy change, 2014	Implementation only, no enforcement, 2015	Early initiation of enforcement, 2016	Continued monitoring, 2017	Main effects		Interaction
						Time	City	Time x City
		Means (SE)				P (df = 3)	P (df = 1)	P (df = 3)
Number of items purchased	Minneapolis	2.2 (0.1)	2.5 (0.1)	2.2 (0.1)	2.4 (0.1)	0.26	**0.008**	0.24
	St. Paul	3.0 (0.2)	2.9 (0.2)	2.6 (0.2)	2.8 (0.4)			
	p-net	**–**	0.10	0.08	0.30			
Total amount spent (US$)^a^	Minneapolis	3.9 (0.3)	4.8 (0.8)	3.5 (0.4)	4.1 (0.4)	0.24	**0.04**	0.87
	St. Paul	5.7 (1.2)	5.7 (1.0)	5.1 (0.9)	5.6 (0.8)			
	p-net	**–**	0.57	0.61	0.40			
Calories purchased^a^	Minneapolis	962 (93)	1179 (184)	838 (112)	928 (114)	**0.01**	**0.02**	0.76
	St. Paul	1421 (221)	1926 (568)	1306 (257)	1630 (455)			
	p-net	**–**	0.33	0.49	0.40			
Energy density^b^	Minneapolis	3.8 (0.1)	3.6 (0.1)	3.6 (0.2)	3.6 (0.1)	0.61	0.99	0.82
	St. Paul	3.6 (0.2)	3.7 (0.1)	3.6 (0.1)	3.5 (0.2)			
	p-net	**–**	0.36	0.50	0.66			
HEI-2010 score (1–100)	Minneapolis	30.7 (0.8)	31.4 (0.7)	30.2 (0.7)	31.3 (0.9)	0.84	0.44	0.69
	St. Paul	29.9 (0.8)	30.5 (1.0)	30.7 (1.1)	30.0 (0.9)			
	p-net	**–**	0.93	0.41	0.75			
Fruit^c^	Minneapolis	1.1 (0.7)	5.0 (1.6)	4.8 (1.9)	4.4 (1.3)	0.08	0.95	0.42
	St. Paul)	2.5 (1.0)	2.9 (1.3)	4.6 (1.8)	3.5 (1.4)			
	p-net	**–**	0.10	0.35	0.19			
Vegetables^c^	Minneapolis	5.1 (1.1)	4.1 (1.3)	1.9 (0.8)	4.4 (1.0)	**0.009**	0.28	0.59
	St. Paul	6.6 (1.8)	4.4 (1.7)	4.3 (1.7)	7.2 (2.3)			
	p-net	**–**	0.72	0.29	0.57			
Whole grains^c^	Minneapolis	7.9 (1.5)	7.4 (1.5)	6.6 (1.4)	7.6 (1.3)	0.94	0.83	0.95
	St. Paul	7.4 (1.4)	8.3 (1.8)	7.4 (1.3)	7.4 (1.5)			
	p-net	**–**	0.66	0.63	0.89			
Skim or reduced fat milk^c^	Minneapolis	4.2 (1.3)	2.3 (0.7)	1.8 (0.8)	1.3 (0.6)	0.62	0.07	0.07
	St. Paul	2.4 (0.9)	4.7 (1.0)	3.1 (1.0)	4.7 (1.1)			
	p-net	**–**	**0.02**	0.10	**0.01**			
Added sugars (% of calories)	Minneapolis	36.5 (2.5)	37.0 (2.0)	42.8 (2.0)	43.7 (2.3)	**0**.**01**	0.89	0.76
	St. Paul	36.3 (2.3)	39.2 (3.1)	41.7 (2.6)	41.5 (3.0)			
	p-net	**–**	0.58	0.84	0.67			
Saturated fatty acids (% of calories)	Minneapolis	8.1 (0.5)	7.8 (0.6)	6.4 (0.5)	6.4 (0.4)	**0.0006**	0.36	0.39
	St. Paul	8.6 (0.5)	8.1 (0.6)	6.1 (0.5)	7.6 (0.6)			
	p-net	**–**	0.75	0.39	0.53			
Sodium^a^ (per 1000 cal)	Minneapolis	1389 (146)	2321 (762)	995 (89)	1378 (243)	0.08	0.79	0.96
	St. Paul	1488 (203)	1099 (97)	1895 (666)	1404 (372)			
	p-net	–	0.80	0.75	0.93			

Note: Models are adjusted for repeated measures over time and for age (the only covariate that was significant in bivariate comparisons between Minneapolis and St. Paul at baseline). Models are linear regression models except specific product categories (fruit, vegetables, whole grains, milk) are logistic regression models; p-net values refer to changes in time*city effect from Time 1 to Time 2, from Time 1 to Time 3, and from Time 1 to Time 4 respectively

^a^Outcome variable was log-transformed due to skewed distribution (Mean and standard error from non-transformed model; *p*-values from log-transformed model)

^b^Beverages removed; only food items from purchases were used to calculate energy density

^c^Percent of purchases with at least one serving

Note: Number of missing values (if any) for each outcome variable at each time point: Time 1: total amount spent = 6; Time 2: total amount spent = 4, energy density = 1; Time 3: total amount spent = 4, energy density = 1; Time 4: total amount spent = 6

Note: HEI score, and the nutrient variables not calculated for purchases where calories = 0

Bolded values denote *p* < 0.05

Finally, Appendices 3 and 4 presents results from the the home-based longitudinal sub-study of frequent shoppers. Table 6 in Appendix 3 shows baseline characteristics of the participants the sub-study. On average, participants were middle-aged, and households included 2 adults and 2 children. The sample was 63% non-White. The only significant difference between participants recruited from Minneapolis versus St. Paul stores was by employment status (e.g., 26% vs. 44% unemployed in Minneapolis and St. Paul, respectively). Table 7 in Appendix 4 details the results from the longitudinal HFI data analyses. Findings show no significant changes over time and/or by city for home food environment indices, specifically overall obesogenicity, vegetable, fruit, reduced fat milk and/or whole grain breakfast cereal availability in the home.

## Discussion

Our findings indicate significant improvements in healthy food availability in food stores in our sample over time in Minneapolis. However, such improvements were also evident in stores in St. Paul and did not differ significantly between the two cities. There were not clear improvements over time in healthy food purchasing in these stores or healthy home food availability among frequent shoppers.

Findings indicate ordinance compliance is a concern, with only 10% of Minneapolis retailers in our sample meeting all ordinance standards in 2017, and only half meeting 8 of 10 standards. We did not observe significant improvement in the percent of stores that were ully compliant between 2014 and 2017 in Minneapolis compared with St. Paul, though the percent of stores meeting at least 8 of 10 ordinance standards in Minneapolis increased from 24 to 51%. In post hoc analyses, we observed improvements in some markers of compliance and healthy food availability in Minneapolis retailers, but improvements were not statistically different from those observed in St. Paul, and could be attributable to underlying trends in the food marketplace.

In considering the limited ordinance compliance to date, additional enforcement may be needed. Deterrence Theory asserts that in order for a policy to be effective, enforcement needs to be both swift and certain [39, 40]. Thus, follow-up inspections need to occur after a violation is issued, and citations need to be written for continued non-compliance. In practice, however, it can be extremely challenging for Minneapolis health inspectors to complete follow-up inspections for staple foods violations due to limited staff capacity. Faced with a growing number of businesses to inspect and an insufficient number of inspectors, ensuring that critical food safety standards are met often becomes the sole priority when deploying staffing resources. (K. Klingler, personal communication).

Overall, our study sample was purposefully selected to represent stores that would be most challenged by the ordinance. As a result, overall city-level retailer compliance was likely higher than what is represented in our data. However, we expected increases in healthy food availability in response to the ordinance to be most likely observed in our sample, given many of these retailers did not meet ordinance standards at the initiation of our study (compared to supermarkets and other large stores, which we expected would have met these standards in 2014, and thus not included in this study).

Despite the four time points for data collection, this research still reflects a relatively early stage of policy enforcement. During policymaking, city leaders added the one-year implementation-only period with no enforcement to the policy timeline between 2015 and 2016; this addition was in response to initial concerns from retailers about whether they would have sufficient time to come into compliance. During this time, Minneapolis Health Department invested in multifaceted communication and technical assistance to retailers. In addition, health department staffers from their Healthy Living Team visited all eligible retailers, conducted store assessments and reviewed ordinance requirements with retailers. Repeated in-person visits were conducted for retailers initially found to be non-compliant.

To our knowledge, the Minneapolis Staple Foods Ordinance is the first policy of its kind requiring retailers to stock specific types and quantities of healthy foods as a condition of licensing. Recently, Passiac, NJ passed a similar Staple Foods policy, [41] and other cities are actively considering similar actions. Despite being highlighted as a model policy for improving healthy food access, [19] our findings underscore challenges with implementation. This is especially important because, to date, policy opportunities to effectively enhance food access in retail settings have been limited. For example, policies incentivizing new supermarkets opening in limited access areas through Healthy Food Financing initiatives in Philadelphia, PA and New York, NY (USA) have shown no consistent effect on residents’ diet-related outcomes 6–12 months after store openings [42, 43]. Another study in Pittsburgh, PA (USA) found the opening of a government-subsidized supermarket had significant effects on several diet-related outcomes, but use of the new supermarket was not associated with diet changes [44]. Other efforts using local zoning in Los Angeles, CA (USA) to restrict the opening or remodeling of stand-alone fast-food restaurants also did not show significant impacts on diet-related outcomes [45]. In sum, these studies illustrate on-going challenges of food access-related policies.

Partly driven by these challenges and mixed findings, there is increasing interest in minimum stocking requirements policies for improving healthy food offerings in small food retailers across the country. In 2016, the Robert Wood Johnson Foundation published a report of expert panel recommendations for healthy stocking criteria for small retail food stores, intended for adoption across a range of programmatic and policy initiatives [46]. Importantly, minimum stocking policies are also featured in retailer authorization in federal nutrition assistance programs. For example, after WIC increased retailer stocking requirements for healthy foods among its 47,000 authorized stores in 2009, but – notably – also changed benefit provisions to allow more healthy foods to be purchased by program participants, many positive outcomes were observed including improvements in healthy food and beverage availability in stores and improved dietary intake among WIC participants [47]. More recently, the USDA considered a rule that would notably enhance the minimum staple foods a retailer must stock in order to be SNAP authorized; however, this rulemaking process had experienced considerable delays and roll-backs. Currently, stocking requirements for SNAP authorized retailers are minimal [48].

Furthermore, additional retail strategies may need to be employed beyond the implementation of stocking standards. Although availability of healthy foods is one critical component to improving healthy purchasing and consumption, a recent report on recommendations for healthy food practices in small stores also emphasized the importance of engaging in additional key marketing strategies in these retail settings [46]. Such strategies include thoughtful placement, advantageous pricing and effective promotion of healthy foods and beverages within the store environment. To our knowledge, however, the peer-reviewed scientific literature lacks the depth to determine a minimum amount of healthy food and beverage marketing needed to change purchasing behaviors in small retail food stores, either alone or in combination with mandatory stocking standards. More research is needed in this area.

Overall, our study has a number of strengths, as well as limitations. Its strengths include robust measurement of food environments of understudied food retailers (small and non-traditional food stores), objective assessments of customer purchasing, and parallel measurement of store environments and customer purchasing from a well-matched control city. Limitations include: (a) it was set in one geographic area, which may limit generalizability, (b) it evaluated impact during a time at which very limited policy enforcement is occurring, (c) these analyses did not examine predictors of compliance, and thus cannot give direct insights into reasons why retailers did or did not comply with the policy, (d) although this study had good overall retailer participation, there appeared to be greater participation among Minneapolis retailers compared to St. Paul retailers and (d) this evaluation was limited to policy impact on food environments and customer purchasing only, and did not assess overall dietary intake of customers. Future work should explore other aspects of policy impact including potential neighborhood disparities in impact on policy implementation, retailers’ perceptions of the policy and its challenges, and factors related to pricing.

## Conclusions

In summary, our study is the first of its kind to evaluate the impact of a local staple foods ordinance. Findings indicate that, in the context of limited compliance, the Minneapolis Staple Foods Ordinance has not led to improvements in the healthfulness of the store environment or the nutritional quality of food purchases in small and non-traditional stores in Minneapolis compared to those in the control city, St. Paul. The finding that compliance has been limited may reflect the challenges and time required for policy implementation.

Despite increasing attention on public health policy interventions, particularly those addressing nutrition and obesity prevention, current policy research in the field tends to focus heavily on health outcomes evaluation, with limited evaluation of policy processes and implementation [49]. Previous research has repeatedly highlighted the challenges faced by small and non-traditional retailers in stocking and selling healthy foods. Although significant efforts were made in developing the Minneapolis Staple Foods Ordinance to ensure the standards were reasonable and achievable even for the smallest food retailers in the city, it appears retailers may still be facing challenges in meeting requirements. More work is needed to understand what these challenges are and how they can be overcome. It is also possible that even retailers who can readily meet the ordinance stocking standards are not doing so, perhaps because of insufficient enforcement. Future work will be needed to continue to monitor policy impact, if and when enforcement improves. This work will allow for a better understanding of the timeline for compliance and whether additional steps are necessary for successful implementation of this staple foods ordinance. Furthermore, effects of the ordinance on the healthfulness of the store environment and the nutritional quality of food purchases in the context of a higher level of compliance may be evaluated through our future work.

## Figures and Tables

**Fig. 2:**
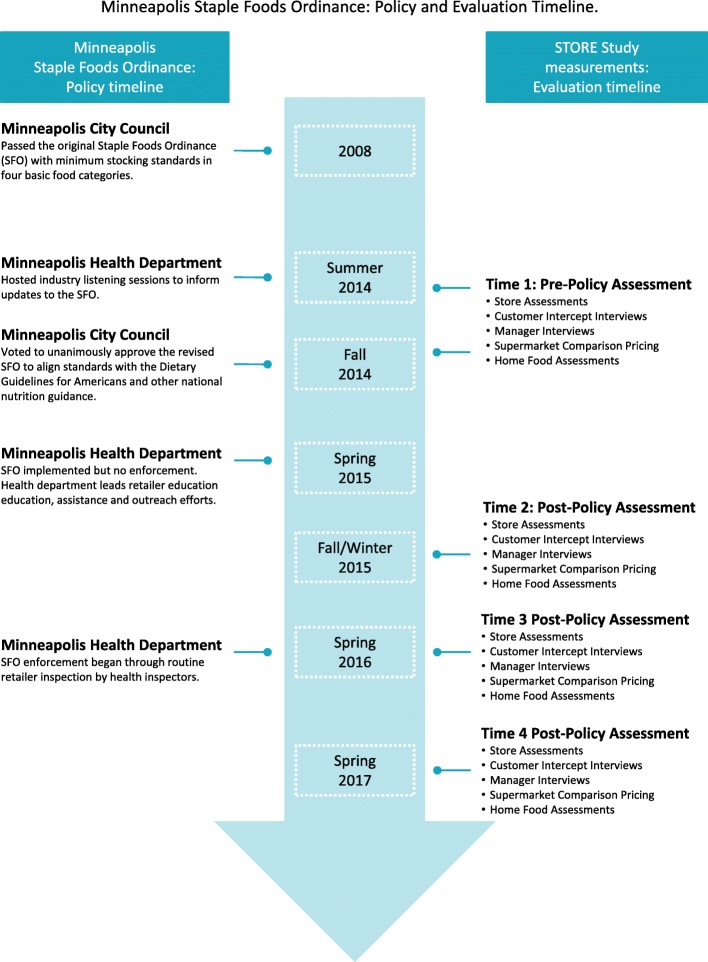
Minneapolis Staple Foods Ordinance: Policy and Evaluation Timeline

## Data Availability

The datasets used for the analyses presented in this manuscript are available online via the Data Repository for the University of Minnesota (https://conservancy.umn.edu/handle/11299/205351). The only data withheld from these datasets (i.e., will not be publicly accessible) are variables detailing (1) retailer type (i.e., convenience store, gas mart, pharmacy, dollar store) and (2) number of aisles in the store. Concerns have been raised about the ability to deduce which retailers participated if these variables were to be included in the dataset, particularly when available in combination with the neighborhood-level data already to be included in the public dataset. These two variables were only used in our descriptive analyses (presented in Table 1), and their omission will not hinder a reader’s ability to replicate the findings from our main models.

## Appendix 3

**Table 6:** Descriptive characteristics of participants during their initial STORE Study home visit (either time 1 or time 2), recruited from Minneapolis and St. Paul stores via customer intercept interviews, 2014–2015 (*n* = 88)

	Recruited from a store in:	
	Minneapolis (*n* = 46)	St. Paul (n = 42)
	Mean (sd)	Mean (sd)
Age (years)	42 (15)	46 (12)
People living in household		
Adults	1.9 (1.0)	2.0 (1.1)
Children	1.7 (1.1)	1.8 (1.1)
	**Percent**	**Percent**
Sex: male	43	33
Race/ethnicity		
Hispanic	7	5
Non-Hispanic		
White	37	37
Black	43	49
American Indian/Alaskan Native	7	5
Asian	0	0
Other race	0	2
Multi-race	7	2
Education		
High school diploma or less	46	55
Some college	37	31
Bachelor’s degree or higher	17	14
Employment		
Employed	**61**	**56**
Unemployed	**26**	**44**
Retired	**13**	**0**
Frequency of purchasing food/drinks at store (during past 30 days)		
At least once a day	17	14
1–6 times a week	50	45
Less than once a week	33	40
Weight status		
Normal (BMI ≥18.5, < 25 kg/m^2^)	16	29
Overweight (BMI ≥ 25, < 30 kg/m^2^)	41	36
Obese (BMI ≥ 30 kg/m^2^)	43	36

Note: Bold text indicates significant chi-square test (p < .05) between cities

Note: 56 participants had their first home visit at Time 1 and 32 had their first home visit at Time 2

Note: Number of missing values (if any) for each variable: age = 2; race/ethnicity = 1; employment = 1; weight status = 2

## Appendix 4

**Table 7:** Impact of the Minneapolis Staple Foods Ordinance on healthy home food availability among frequent shoppers, 2014–2017 (n = 88)

Outcome	City	Assessment Period				Overall effects		
		Time 1	Time 2	Time 3	Time 4	Main effects		Interaction
		Pre-policy revision, 2014	Implementa-tion only, 2015	Early initiation of enforcement, 2016	Continued monitoring, 2017	Time	City	Time x City
		Means (SE)				P (df = 3)	P (df = 1)	P (df = 3)
Obesogenicity home food availability score	Minneapolis	16.7 (1.7)	14. 8 (1.4)	16.5 (1.7)	16.6 (1.2)	0.30	0.85	0.52
	St. Paul	15.9 (1.5)	16.2 (1.5)	16.0 (1.4)	17.8 (1.4)			
	p-net		p = 0.27	*p* = 0.82	p = 0.32			
Types of vegetables in the home	Minneapolis	7.4 (0.7)	6.2 (0.6)	6.5 (0.6)	7.9 (0.5)	0.27	0.27	0.07
	St. Paul	7.2 (0.7)	7.8 (0.6)	8.0 (0.5)	7.6 (0.6)			
	p-net		*p* = 0.04	*p* = 0.09	*p* = 0.87			
Types of fruit in the home	Minneapolis	4.5 (0.6)	3.7 (0.6)	4.2 (0.6)	4.5 (0.6)	0.09	0.64	0.78
	St. Paul	4.6 (0.8)	4.0 (0.6)	4.1 (0.7)	5.1 (0.7)			
	p-net		p = 0.87	p = 0.87	*p* = 0.59			
% of participants with ≥1 reduced fat milk product in the home	Minneapolis	70.0 (9.7)	67.5 (7.6)	66.0 (6.9)	72.9 (6.9)	0.97	0.81	0.89
	St. Paul	73.1 (8.8)	71.2 (7.9)	70.7 (8.3)	68.6 (9.2)			
	p-net		p = 0.98	*p* = 0.93	*p* = 0.60			
% of participants with ≥1 whole grain breakfast cereal in the home	Minneapolis	70.8 (8.8)	75.8 (6.9)	76.9 (7.3)	72.4 (6.9)	0.96	0.46	0.72
	St. Paul	69.3 (9.3)	68.9 (8.5)	63.9 (8.5)	73.0 (8.5)			
	p-net		*p* = 0.63	p = 0.43	p = 0.90			

Note: Data are from regression models adjusted for repeated measures over time and for employment status (the only variable that was significantly different in bivariate comparisons between Minneapolis (policy) and St. Paul (control) at the first study home visit); p-net values refer to changes in time*city effect from Time 1 to Time 2, Time 1 to Time 3, and Time 1 to Time 4, respectively

Note: No missing data at any time point
